# FW: An R Package for Finlay–Wilkinson Regression that Incorporates Genomic/Pedigree Information and Covariance Structures Between Environments

**DOI:** 10.1534/g3.115.026328

**Published:** 2015-12-29

**Authors:** Lian Lian, Gustavo de los Campos

**Affiliations:** * Department of Epidemiology and Biostatistics, Michigan State University, East Lansing, Michigan 48824; † Department of Probability and Statistics, Michigan State University, East Lansing, Michigan 48824

**Keywords:** Bayesian, Finlay–Wilkinson, genomic/environment, correlation, genotype by environment interaction, reaction norm, GenPred, genomic selection, shared data resource

## Abstract

The Finlay–Wilkinson regression (FW) is a popular method among plant breeders to describe genotype by environment interaction. The standard implementation is a two-step procedure that uses environment (sample) means as covariates in a within-line ordinary least squares (OLS) regression. This procedure can be suboptimal for at least four reasons: (1) in the first step environmental means are typically estimated without considering genetic-by-environment interactions, (2) in the second step uncertainty about the environmental means is ignored, (3) estimation is performed regarding lines and environment as fixed effects, and (4) the procedure does not incorporate genetic (either pedigree-derived or marker-derived) relationships. Su *et al.* proposed to address these problems using a Bayesian method that allows simultaneous estimation of environmental and genotype parameters, and allows incorporation of pedigree information. In this article we: (1) extend the model presented by Su *et al.* to allow integration of genomic information [*e.g.*, single nucleotide polymorphism (SNP)] and covariance between environments, (2) present an R package (FW) that implements these methods, and (3) illustrate the use of the package using examples based on real data. The FW R package implements both the two-step OLS method and a full Bayesian approach for Finlay–Wilkinson regression with a very simple interface. Using a real wheat data set we demonstrate that the prediction accuracy of the Bayesian approach is consistently higher than the one achieved by the two-step OLS method.

Plant breeders use the Finlay–Wilkinson regression (Finlay and Wilkinson 1963) to assess stability of varieties across different environments. The FW aims at assessing how the expected performance of a genotype varies as a function of the environmental effects. Usually this is achieved by regressing the performance of each genotype on the environmental means. Compared with a completely unstructured genotype by environment interaction (G × E) model that fits every level of genotype and environment combination, the Finlay–Wilkinson regression is parsimonious and can reveal a trend of variety performance across environments. Breeders can use this model to select for plants either based on stability or on responsiveness to environment potential (Walsh and Lynch 2014).

The standard implementation of Finlay–Wilkinson regression is a two-step procedure whereas in the first step environmental sample means are computed and in the second step intercepts and slopes of each line are estimated by regressing, within line, the performance of each line on the estimated environmental means. This procedure has at least four potential limitations: (1) in the first step environmental means are typically estimated without considering G × E; (2) in the second step, uncertainty about the environmental means is ignored; (3) the environmental means and the variety intercepts and slopes are regarded as fixed effects (this can lead to large sampling variance of estimates); and (4) the procedure does not offer a clear way of incorporating pedigree or molecular marker information when estimating the intercepts and slopes of the lines. These drawbacks can induce biases (especially in incomplete designs where a few lines are evaluated in each environment) and lead to large sampling variance of estimates.

Su *et al.* (2006) proposed a Bayesian method that addresses the limitations of the standard two-step procedure. The methodology described by Su *et al.*: (1) uses a Gibbs sampler that allows estimating environmental and genotype parameters jointly; (2) fully accounts for confounding and uncertainty about environmental means; (3) treats environmental means and the intercepts and slopes of the lines as random – this treatment usually performs better than ordinary least squares in terms of mean-squared error and of prediction accuracy, especially when the number of parameters to be estimated is large relative to sample size (Copas 1983; Frank and Friedman 1993); and (4) allows incorporating pedigree information into the model. Using simulations, Su *et al.* (2006) reported better statistical performance of the Bayesian method for estimating model parameters. In this article we extend the model proposed by Su *et al.* (2006) in ways that allow incorporating genomic [*e.g.*, single nucleotide polymorphism (SNP)] information and covariance between the environment effects.

To the best of our knowledge the methodology described by Su *et al.* for animal breeding applications has not been considered in plant breeding, and there is no publicly available user-friendly software for implementing a Bayesian Finlay-Wilkinson regression. Therefore, in this article we introduce an R package (R Development Core Team 2011) that implements the Finlay–Wilkinson regression. The FW package implements both the two-step ordinary least squares (OLS) procedure and Bayesian single step procedure that allows incorporating covariance structure for varieties (*e.g.*, a pedigree or marker-derived kinship matrix) and environments. We describe the methods implemented in the package and show with examples how this package can be used to perform the Finlay–Wilkinson regression with both methods. Finally, we present an evaluation of prediction accuracy for the Bayesian and two-step OLS methods with a wheat data set.

## Model Specification and Algorithm

In a reaction norm model (Gregorius and Namkoong 1986; Perkins and Jinks 1968) the phenotypic record of the *k*^th^ replicate of the *i*^th^ variety observed in the *j*^th^ environment is modeled as follows:

$$y_{ijk}=\mu +g_{i}+h_{j}+b_{i}h_{j}+\varepsilon_{ijk}$$ [Equation 1]

where $g_{i}$ is the main effect of *i*^th^ variety and $h_{j}$ is the main effect of the *j*^th^ environment, and $\varepsilon_{ijk}$ is an error term, usually assumed to be IID normal with mean zero and variance $\sigma_{\varepsilon }^{2}$. When we reorganize Equation 1 into the form: $y_{ijk}=\mu +g_{i}+(b_{i}+1)h_{j}+\varepsilon_{ijk}$, we can recognize that $b_{i}+1\;$is the change of expected variety performance per unit change of the environment effect ($h_{j}$). If there are no replicates the index *k* can be removed. With this, the equation reduces to $y_{ij}=\mu +g_{i}+h_{j}+b_{i}h_{j\;}+\varepsilon_{ij}$. The collection of parameters to be estimated from the model of Equation 1 includes the intercept and the vectors of effects: $g=\{{\text{g}}_{\text{i}}\}$, $b=\{{\text{b}}_{\text{i}}\}$, and $h=\{{\text{h}}_{\text{j}}\}$.

### Estimation using two-steps methods

The Finlay–Wilkinson regression requires regressing the observed phenotypes of the line on environment effects. In the standard Finlay–Wilkinson regression (Finlay and Wilkinson 1963) the environmental effects are computed from the sample environmental means. However, in incomplete designs the sample mean of an environment may not be an unbiased estimate of the true environment mean. Therefore, a better estimate of environment effects comes from a regression that accounts for both environment effects and genotype effects, that is:

 **Step 1**– estimate the environmental effect using a main effects model:
  $$y_{ijk}=\mu +g_{i}+h_{j}+\varepsilon_{ijk}$$ [Equation 2]

The above regression yields estimates of environment effects (${\hat{h}}_{j}$); these can be used in the second step to estimate the intercepts and slopes of each line.

 **Step 2**– replace $h_{j}$ with ${\hat{h}}_{j}$ in Equation 1 yielding:
  $$y_{ijk}=\mu +g_{\text{i}}+{\hat{h}}_{j}+b_{i}{\hat{h}}_{j}+\varepsilon_{ijk}$$ [Equation 3]

Both Equation 2 and Equation 3 can be implemented with either OLS or mixed models. The current FW package implemented both Step 1 and Step 2 with OLS. In Step 1, Equation 2 is fitted with the constraint that $\underset{j}{\sum }{\hat{h}}_{j}=0$ and $\underset{i}{\sum }{\hat{g}}_{i}=0$. Step 2 is implemented by fitting Equation 3 separately within each line with the constraint $\hat{\mu }=0$.

### Bayesian approach

Bayesian inferences are based on the posterior distribution of unknown parameters given the data: p(θ|y)∝p(y|θ)p(θ), where ***θ*** represents the collection of the unknowns: θ={μ, ***g***, ***b***, ***h***, $\sigma_{g}^{2}$, $\sigma_{b}^{2}$, $\sigma_{h}^{2}$, $\sigma_{\varepsilon }^{2}\}$, p(y|θ) is the conditional distribution of the data given the parameters, and p(θ) is the joint prior distribution assigned to the model unknowns. According to Equation 1 and assuming IID normal residuals, we have:

$$p(y|\theta )=\underset{ijk}{\prod }\text{N}(\mu +g_{i}+h_{j}+b_{i}h_{j},\sigma_{\varepsilon }^{2})$$.

In the FW package, the prior density is assumed to have the following form: $p\left(\theta \right)=p\left(\sigma_{\varepsilon }^{2}\right)p\left(g|\sigma_{g}^{2}\right)p\left(b|\sigma_{b}^{2}\right)p\left(h|\sigma_{h}^{2}\right)p(\sigma_{g}^{2})p(\sigma_{b}^{2})p(\sigma_{h}^{2})$. The residual variance $\sigma_{\varepsilon }^{2}$ is assigned a scaled-inverse $\chi^{2}$ distribution: $\sigma_{\varepsilon }^{2}∼\chi^{-2}(\nu_{\varepsilon },S_{\varepsilon }^{2})$, with degrees of freedom $\nu_{\varepsilon }$ (>0) and scale parameter $S_{\varepsilon }^{2}$ (>0), in the parameterization used $E[\sigma_{\varepsilon }^{2}]=\frac{\nu_{\varepsilon }S_{\varepsilon }^{2}}{\nu_{\varepsilon }-2}$. The overall mean *μ* is assigned a flat prior. The prior distributions for ***g***, ***b***, and ***h*** are all multivariate normal: $h∼\text{N}(0,\;H\sigma_{h}^{2})$, **$g∼\text{N}(0,A\sigma_{g}^{2})$**, $b∼\text{N}(0,A\sigma_{b}^{2})$, where **H** is a covariance structure describing covariances between the environment effects (this can be a covariance structure based on spatial information) and **A** is a covariance structure describing covariances between levels of the random effects **g** and **b** (**A** could be either a pedigree or marker-derived relationship matrix). Independence between the effects of the levels of any of the random effects can be obtained by setting either **A** or **H** to be an identity matrix. The variance components $\sigma_{h}^{2}$, $\sigma_{g}^{2}$, and $\sigma_{b}^{2}$ are assigned scaled-inverse-$\chi^{2}$ distributions whose shape are controlled by variance-specific degree of freedom and scale hyper-parameters. The FW package offers users the possibility of specifying hyper-parameters (degree of freedom and scale parameters); however, if these are not specified, specific sets of rules similar to those described in Pérez *et al.* (2010) are used to determine those parameters. Further details about this are given in Supporting Information, File S2.

In the model described above the posterior density does not have a closed form; however, estimates of features of the posterior distribution (*e.g.*, posterior means, posterior standard deviations, or credibility regions) can be derived using Monte Carlo methods. The FW package draws samples from the posterior distribution of the model using a Gibbs sampler (Casella and George 1992; Geman and Geman 1984) similar to the one described in Su *et al.* (2006); details of the implementation of Gibbs sampler are provided in File S1.

## Software

The FW package implements both a two-step OLS method and the Bayesian method described in the previous section. Typing the following command in R will install the package:

**Table:** 

library(devtools)
install_github(“lian0090/FW”)

### Wheat data set

The package includes a data set that can be used to run examples. The data set [originally made publicly available by Crossa *et al.* (2010)] contains data for 599 wheat lines from CIMMYT’s Global Wheat Program evaluated for grain yield in four environments. The data set becomes available in the R environment by running the following R-code:

**Table:** 

library(FW)
data(wheat)

Function library() loads the package, and data() loads data sets included in the package into the environment. The above code loads the following objects into the environment: (1) wheat.Y, a data.frame (2396 × 3) containing the grain yield (average of two plot records, $y) of 599 wheat lines ($VAR) in four environments ($ENV) and (2) wheat.G (599 × 599) is a genomic relationship matrix computed from DArT markers. Further details about this data set can be found in Crossa *et al.* (2010).

### User interface

All the arguments of the FW function have default values, except the response variable and the corresponding identifiers for varieties and environments. A basic call to the FW program is given in Box 1.

**Box 1 Basic call of the FW function**

**Table:** 

1	library(FW)
2	data(wheat)
3	attach(wheat.Y)
4	lm1 = FW(y = y,VAR = VAR,ENV = ENV,method=”OLS”)
5	lm2 = FW(y = y,VAR = VAR,ENV = ENV)

When the call of the FW function is done using the code in line 4 of Box 1, FW fits a Finlay–Wilkinson regression with the two-step OLS method: y (numeric, *n*, NAs are allowed) is the response variable, VAR (character, *n*, NAs are not allowed) are the identifiers for the varieties which are treated as labels; ENV (character, *n*, NAs are not allowed) are the identifiers for the environments; method is used to describe what method to use: “OLS” for ordinary least squares. The default method (“Gibbs”) is the Bayesian regression; this can be invoked using the code in line 5 of Box 1. By default, a single chain of Gibbs sampler is run with a total of 5000 cycles and the samples from the first 3000 cycles are used for Burn-in, and samples from the remaining 2000 cycles for inference (the user is advised to run longer chains and to check convergence as well as the size of Monte Carlo errors). The FW function provides many additional arguments that can be used to specify the model (*e.g.*, providing covariance matrices for varieties and environments, user-defined values for hyperparameters) and the algorithm (number of chains, number of iterations, *etc*.); details can be found in the user manual and in the examples presented below.

After fitting either OLS or Gibbs method, FW function returns a list with estimates and arguments used in the call; a brief description of the outputs follows.

### Return

Box 2 shows the structure of the object returned after calling the FW function with the default Gibbs method (see line 1 of Box 2). The first element $y of the list is the response vector used in the call to FW, $whichNa gives the position of the entries in y that were missing, $mu (vector), $g (matrix), $b (matrix), $h (matrix) are the estimated posterior means of *μ*, **g**, **b**, and **h**; $yhat (matrix) is the estimated posterior means of the predictor $\hat{y}$: ${\hat{y}}_{ijk}=\hat{\mu }+{\hat{g}}_{i}+{\hat{h}}_{j}+{\hat{b}}_{i}{\hat{h}}_{j}$; $SD.mu (vector), $SD.g (matrix), $SD.b (matrix), $SD.h (matrix), and $SD.yhat are the estimated posterior standard deviation for *μ*, **g**, **b**, **h**, and **$\mu +g_{i}+h_{j}+b_{i}h_{j}$**, respectively.

With the OLS method, $g, $b, $h, and $yhat all have only one column; with the Gibbs method each column provides estimates derived from one chain of Markov chain Monte Carlo (MCMC). Since the default behavior is to run only one chain the outputs in Box 2 contain only one column; however, if multiple chains are run, estimates from different chains are provided in different columns.

**Box 2 Structure of the object returned by FW**

**Table:** 

1	str(lm2)
2	List of 24
3	$ y: num [1:2396] 6.17 3.14 2.74 3.26 4.99 ...
4	$ whichNa: int(0)
5	$ VAR: chr [1:2396] “775” “775” “775” “775” ...
6	$ ENV: chr [1:2396] “1” “2” “4” “5” ...
7	$ mu: Named num 4.64
8	$ SD.mu: Named num 0.0979
9	$ g: num [1:599, 1] -0.476 0.16 -0.611 ...
10	$ SD.g: num [1:599, 1] 0.224 0.219 0.224 0.208 ...
11	$ b: num [1:599, 1] 0.1604 -0.1255 0.251 ...
12	$ SD.b: num [1:599, 1] 0.237 0.236 0.235 0.24 ...
13	$ h: num [1:4, 1] 0.519 -0.186 -0.776 -1.383 ...
14	$ SD.h: num [1:4, 1] 0.096 0.0999 0.0999 0.103 ...
15	$ yhat: num [1:2396, 1] 5.17 4.3 3.56 2.81 5.21 ...
16	$ SD.yhat: num [1:2396, 1] 0.283 0.217 0.25 0.343 ...
17	$ var_e: Named num 0.3
18	$ SD.var_e: Named num 0.0111
19	$ var_g: Named num 0.0885
20	$ SD.var_g: Named num 0.0116
21	$ var_b: Named num 0.0973
22	$ SD.var_b: Named num 0.0132
23	$ var_h: Named num 0.926
24	$ SD.var_h: Named num 0.595

The outputs $var_e, $var_g, $var_b, and $var_h are the estimated posterior means for $\sigma_{\varepsilon }^{2}$, $\sigma_{g}^{2}$, $\sigma_{b}^{2}$, and $\sigma_{h}^{2}$ (only available for the Gibbs method). Each element of $var_e, $var_g, $var_b, and $var_h correspond to estimates derived from different chains; $SD.var_e, $SD.var_g, $SD.var_b, and $SD.var_h are the estimated posterior standard deviation for $\sigma_{\varepsilon }^{2}$, $\sigma_{g}^{2}$, $\sigma_{b}^{2}$, and $\sigma_{h}^{2}$, respectively.

### Output files

No output files are generated for the OLS method. For the Gibbs method, samples for $\sigma_{\varepsilon }^{2}$, $\sigma_{g}^{2}$, $\sigma_{b}^{2}$, $\sigma_{h}^{2}$, and (by default) the first two elements of **g, b,** and **h** will be saved; as the Gibbs sampler collects samples, these samples are saved to the hard drive (only the most recent samples are retained in the memory); by default, a thinning of 5 is used. Once the iteration process finishes, FW will read all the saved samples into a mcmc object, save the mcmc object into a file samps.rda, and remove the raw sample files. To prevent overloading the RAM with samples by default FW only save samples of the first two entries of the vectors of random effects; however the user can change this behavior by specifying which entries of the vectors are desired using the saveVAR (for **g** and **b**) and saveENV (for **h**) argument. These samples produced by FW can be used to assess convergence and to estimate Monte Carlo standard errors. The file samps.rda can be directly loaded into R using load(’samps.rda’). Once the object containing the samples is loaded in the R environment, the package coda (Plummer *et al.*, 2006) can be used to obtain plots of the chains and compute convergence diagnostics.

## Application examples

In this section we illustrate via examples some of the features of the FW package. Example 1 illustrates how the package can be used to fit Finlay–Wilkinson regression by the OLS method and Gibbs method with and without covariance structure and Example 2 describes how the package can be used for cross-validation analyses. Additional examples involving fine-tuning the Gibbs method (*e.g.*, hyperparameter setup, fitting more than two chains, specify saved samples) are provided in File S2.

### Example 1: fitting models with default setup for 599 wheat lines

Box 3 shows the code used to fit a FW regression using three different approaches: (1) a two-step OLS method (code in line 3), (2) a Bayesian FW regression assuming independence of lines and of environments (code in lines 3–5), and (3) a Bayesian FW regression that incorporates genomic information (lines 6–8). In the Bayesian models, the seed for the random number generator can be specified using the argument seed (see lines 3–8) and the argument saveAt can be used to add a path and a prefix to be appended to ’samps.rda’ file.

**Box 3 Fit models by default parameters**

**Table:** 

1	library(FW); data(wheat); attach(wheat.Y)
2	OLS = FW(y = y,VAR = VAR,ENV = ENV, method=”OLS”)
3	GibbsI = FW(y = y,VAR = VAR,ENV = ENV,
4	method=”Gibbs”,seed = 12345,saveAt=”GibbsI”,nIter = 50000
5	,burnIn = 5000)
6	GibbsA = FW(y = y,VAR = VAR,ENV = ENV,
7	method=”Gibbs”,A = wheat.G,seed = 12345,
8	saveAt=”GibbsA”,nIter = 50000,burnIn = 5000)
9	load(“GibbsIsamps.rda”)
10	HPDinterval(samps[,c(“var_e”,”var_g”,”var_b”,”var_h”)])
11	load(“GibbsAsamps.rda”)
12	HPDinterval(samps[,c(“var_e”,”var_g”,”var_b”,”var_h”)])

Parameter estimates (estimated posterior means) can be directly extracted from the FW object as illustrated in Box 2. Other features of the posterior distribution (*e.g.*, 95% credibility intervals for the parameters) can be obtained by *post hoc* analyses of the samples included in the rda file generated by the program (see lines 9–12 of Box 3). In Table 1, we listed the estimates of variance components from the three models. For the OLS method, only the residual variance $\sigma_{\varepsilon }^{2}$ (weighted mean of residual variance for each within-line regression by its residual degree of freedom) is estimated. The estimated error variances are very similar across the three models. Also from Table 1, we can see that the estimated variance of the main effects of the environments is large relative to both the error variance and the phenotypic variance.

**Table 1:** Estimated variance components (posterior 95% credibility intervals in parentheses) from different models

Parameters	FW Output	OLS	GibbsI (A = I)	GibbsA (A = G)
$\sigma_{\varepsilon }^{2}$	$var_e (Gibbs) $var_e_weighted(OLS)	0.32	0.30 (0.28, 0.32)	0.30 (0.28, 0.32)
$\sigma_{g}^{2}$	$var_g	NA	0.09 (0.07, 0.11)	0.11 (0.08, 0.14)
$\sigma_{b}^{2}$	$var_b	NA	0.10 (0.07, 0.12)	0.13 (0.10, 0.17)
$\sigma_{h}^{2}$	$var_h	NA	0.90 (0.24, 1.90)	0.88 (0.24, 1.88)

The fitness of the models can be examined by the correlations between the observed values y and the fitted values $\hat{y}$ (line 1 of Box 4). The OLS model fitted the data better than both GibbsI and GibbsA: the correlation was 0.91 for the OLS method, 0.88 for GibbsI, and 0.86 for GibbsA.

**Box 4 Correlation between *y* and $\hat{y}$, and correlations for $\hat{b}$ among different models**

**Table:** 

1	cor(y,OLS$yhat);cor(y,GibbsI$yhat);cor(y,GibbsA$yhat);
2	cor(OLS$b,GibbsI$b);cor(OLS$b,GibbsA$b);cor(GibbsI$b,GibbsA$b)

In Table 2, we listed the correlations among parameter estimates from different models (code for $\hat{b}$ was provided in line 2 of Box 4), and noticed that correlations among parameter estimates from different models are high; this is expected considering that the data comes from a full factorial design where all lines are evaluated in all environments.

**Table 2:** Pearson’s product-moment correlation between parameter estimates derived by each of the three methods implemented in Box 3

	OLS–GibbsI	OLS–GibbsA	GibbsI–GibbsA
$\hat{h}$	1.00	1.00	1.00
$\hat{b}$	0.94	0.81	0.83
$\hat{g}$	0.98	0.79	0.81
$\hat{y}$	0.96	0.94	0.97

**Figure 1:**
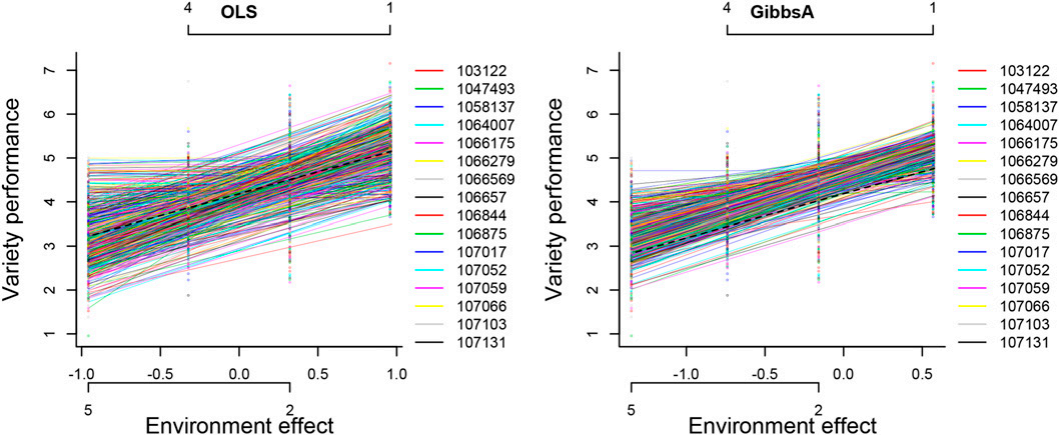
Plot of variety performance *vs.* estimated environment values. Each line represents a different variety. Lines are fitted values and points are the cell means of genotype and environment combination. The horizontal axis displays the estimated environmental effects. The labels of these environments are also displayed; these labels can be removed by setting ENVlabel = F.

The pattern of variety performance in different environments can be visualized by plotting the observed and fitted values against the estimated environment effects. Figure 1 was generated by the calling of plot function in line 2–3 of Box 5. Each line in this plot corresponds to a genotype. The comparison of the results from the OLS and GibbsA reveals interesting patterns: the OLS method predicts a much stronger extent of variability in intercepts and slopes (this is likely due to overfitting, see Example 2 below) than the Bayesian method. The Bayesian method yields ‘smoother’ predictions; this is a direct consequence of the shrinkage-toward-the-mean induced in the Bayesian method by treating effects as random and the use of correlations between genotypes (*e.g.*, genomic relationships).

**Figure 2:**
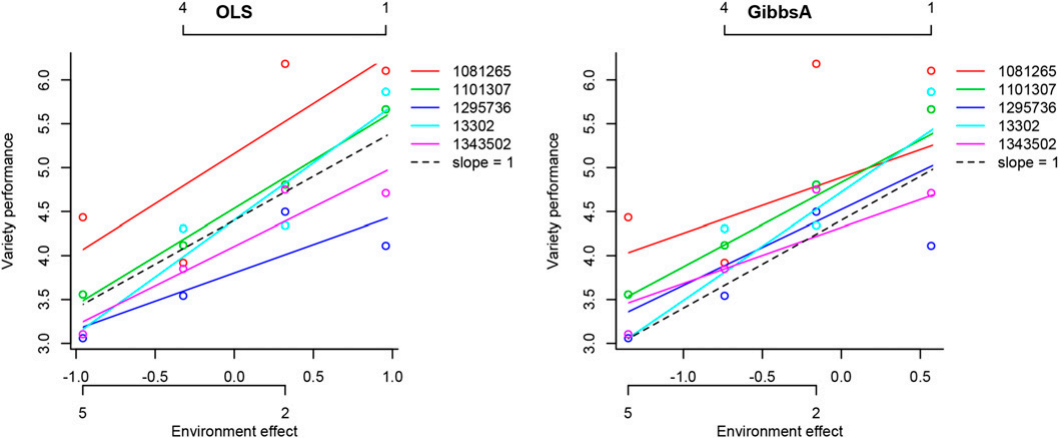
Plot of the performance of five varieties on estimated environment values. Each color represents a different variety. Lines are fitted values and circles are the cell means of genotype by environment combination. The horizontal axis displays the estimated environmental effects. The labels of these environments are also displayed; these labels can be removed by setting ENVlabel = F.

The function plotVAR also allows users to display the curves for a few genotypes (see code in lines 5–9 of Box 5). Using this feature we display in Figure 2 the estimated regressions for five varieties. The slope in the plot corresponds to $1+b_{i}$ and the dashed line corresponds to a slope equal to 1 ($b_{i}=0$); recall that $1+b_{i}$ represents the expected change in performance of the i^th^ variety per unit change in the environment effect. We observe from Figure 2 that line ID = 1081265 performs well in all environments and line ID = 13302 is better adapted to good environments.

### Fitting models with covariance between the environment effects

Covariance structures can be used to induce borrowing of information between levels of a random effect. For instance, pedigree-based or genomic-derived relationships can be used to induce borrowing of information between genotypes. Similarly a covariance structure between the environment effects could be used to induce borrowing of information between environments. Such covariance structures can be derived from previous knowledge about the correlation of the average performance of genotypes in pairs of environments or by using environmental covariates, as demonstrated in Jarquín *et al.* (2014). The FW package allows incorporating covariance between the environment effects; an example of how this can be done is given in Box 6. In this example we compare three analyses. The first model (GibbsI) assumes that the environment effects are independent; this model was fitted previously using the code in Box 3. Subsequently, we modified this model by incorporating a covariance structure that assumes a covariance of 0.9 between environments 1 and 2, and null covariance among the other pairs of environments. This model was fitted using the entire data set (GibbsH) and after setting to NA all the records from the 2nd environment (GibbsH_NA).

**Box 5 Plot fitted models**

**Table:** 

1	par(mfrow = c(1,2))
2	plot(OLS,main=”OLS”, cex = 0.2,lwd = 0.2)
3	plot(GibbsA,main=”GibbsA”, cex = 0.2,lwd = 0.2) #cex controls point
4	#size, lwd controls the line width
5	plot(OLS, plotVAR = c(“1081265”,”1101307”,
6	“1295736”, “13302”, “1343502”), main=”OLS”)
7	plot(GibbsA, plotVAR = c(“1081265”,”1101307”,
8	“1295736”, “13302”, “1343502”),
9	main=”GibbsA”)

Table 3 displays the estimated environment effects derived from each of the analyses. The estimated environment effects derived from GibbsI and GibbsH were almost identical. This happens because in these two examples the data available for each environment dominate over the prior distribution (which in case of GibssH assumes that the effects of environments 1 and 2 are highly correlated). However, when we set to NA all the entries of environment 2 (GibbsH_NA), the estimated effects for environments 1 and 2 are very close. This was entirely driven by the covariance structure H. An intermediate situation can emerge where one environment has records for a few genotypes. In such cases, nondiagonal covariance structures (H) may be used to borrow information between environments.

**Table 3:** Estimated environment effects from GibbsI and GibbsH

ENV	GibbsI	GibbsH	GibbsH_NA
1	0.52	0.51	0.78
2	−0.18	−0.19	0.74
4	−0.78	−0.78	−0.52
5	−1.38	−1.39	−1.11

**Box 6 Including covariance matrix (H) for environments in FW**

**Table:** 

1	H = diag(1,4)
2	H[1,2]=H[2,1]=0.9
3	colnames(H)=rownames(H)=c(1,2,4,5)
4	GibbsH = FW(y = y,VAR = VAR,ENV = ENV, method=“Gibbs”,H = H,seed =
5	12345,nIter = 50000,burnIn = 5000)
6	yNA = y
7	yNA[which(ENV==2)]=NA
8	GibbsH_NA = FW(y = yNA,VAR = VAR,ENV = ENV, method=“Gibbs”,H = H,seed =
9	12345,nIter = 50000,burnIn = 5000)
10	round(cbind(GibbsI$h,GibbsH$h,GibbsH_NA$h),2)

Finally, the example provided by GibbsH_NA also illustrates how H allows predictions to be made about environments without records; if such environments are correlated with other environments for which we have data, in principle we can infer the effects for those environments. This of course will not be possible if H is diagonal.

### Assessment of convergence for Bayesian FW regressions

**Figure 3:**
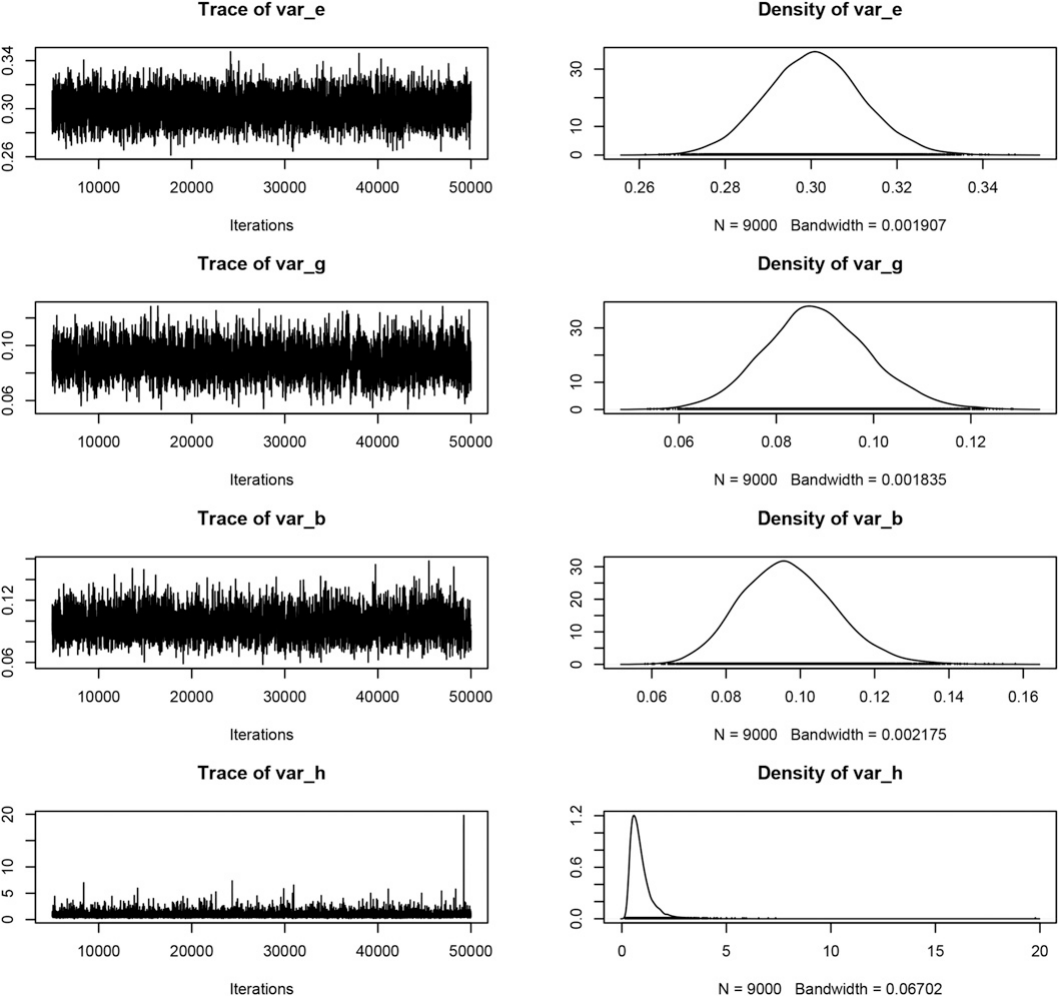
Trace and density plot of variance components from GibbsA.

The convergence of Gibbs sampler can be examined by plotting the samples collected by FW. The code in Box 7 illustrates how to produce trace plots: lines 1–2 load and plot the samples from GibbsI and lines 3–4 do the same for GibbsA. Mixing was reasonably good (samples traverse through the sample space in relatively few steps, and can be verified by low average autocorrelation between samples: for example the average autocorrelation was 0.05 for var_e at lag 5, see line 5 of Box 7) in both cases for the variance components [$\sigma_{\varepsilon }^{2}$ (var_e), $\sigma_{g}^{2}$ (var_g), $\sigma_{b}^{2}$ (var_b)], genotype main effects **g** (g), genotype slope **b** (b), and the function predictor $\hat{y}\;$(yhat). There are many high peaks in the trace plot of $\sigma_{h}^{2}$ (var_h), which indicates that the distribution of $\sigma_{h}^{2}$ is skewed (this is also self-evident in the density plot). This should be expected since there are only four levels of environment effect and scaled-inverse chi-square distribution with few degrees of freedom is highly skewed. Figure 3 reproduces the trace plot of the variance components (var_e, var_g, var_b, var_h).

**Figure 4:**
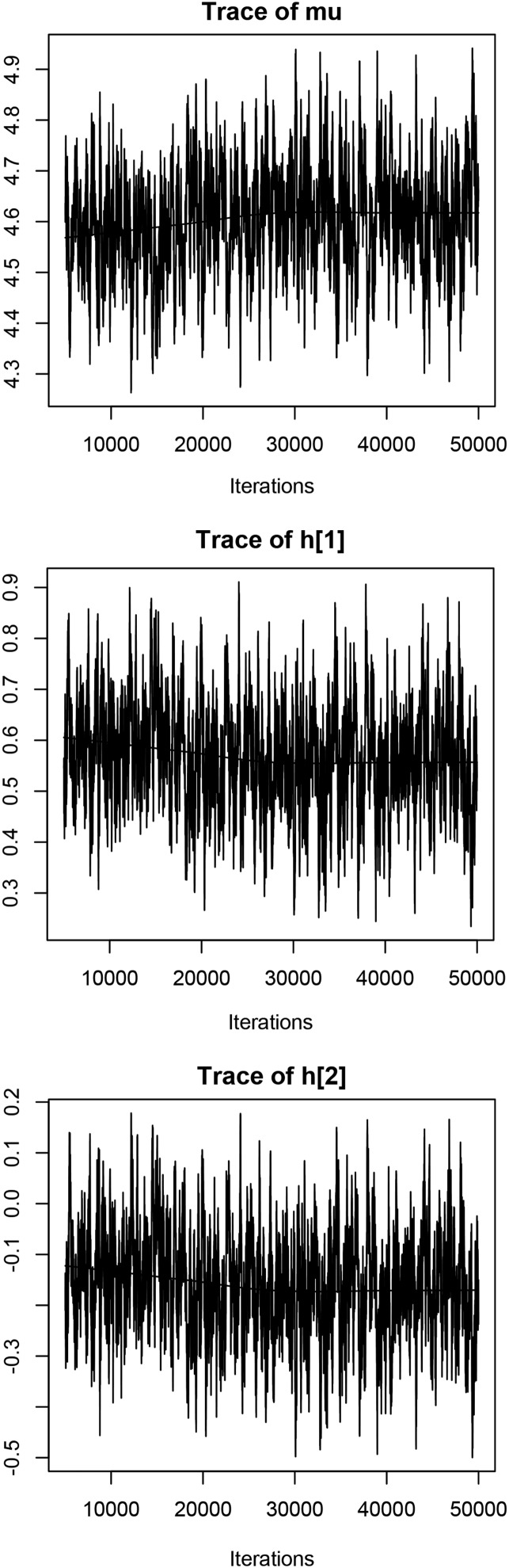
Trace plot of the intercept (mu) and the first two levels of environment effects (h[1]) and h[2]) from GibbsA.

The mixing for the intercept *μ* and the environment effects (the entries of **h**) can be slow in multiplicative models (*e.g.*, Shariati *et al.*, 2009). Therefore, the user is advised to check convergence to the posterior distribution and the magnitude of Monte Carlo standard errors. Convergence to the posterior distribution can be assessed graphically using a trace plot for single or more formally multiple chains. Figure 4 reproduces the trace plot for intercept *μ* and the first two elements of environment effect, h[1] and h[2]; in all cases we used samples from model GibbsA. From Figure 4, we can see that even the mixing of h[1] and h[2] is slow; when running 50,000 iterations, the chain has converged to relative constant sample means. The Time-series standard error for the sample means of h[1] and h[2] are both around 0.0065, which is at a reasonable level (obtained by line 6 of Box 7). An example of how to assess convergence using multiple chains is provided in File S2.

**Box 7 Plot of Gibbs samples**

**Table:** 

1	load(“GibbsIsamps.rda”)
2	plot(samps,ask = T)
3	load(“GibbsAsamps.rda”)
4	plot(samps,ask = T)
5	autocorr(samps[[1]][,”var_e”])
6	summary(samps)

### Example 2: assessment of prediction accuracy in testing data sets

Example 1 suggests that the OLS method fitted the training data better than the Bayesian models; this is expected because shrinkage reduces fitness to the data used to train a model. However, better model fitness does not necessarily imply higher prediction accuracy in validation data sets. In the following example we illustrate how to use the FW for assessment of prediction accuracy using cross-validation.

To assess the ability of different models for predicting new data, we modified the code in Box 3 by setting NA to randomly selected entries of the phenotypic vector (*i.e.*, one record out of four per line was randomly selected and labeled as NA; see code in lines 1–5 in Box 8). The FW package produces estimates and predictions for all the lines, environments, and entries of the phenotypic vector, including those that had observed values and those that had NA. Therefore, predictions for entries with masked phenotypes can be used to assess prediction accuracy in validation data sets (see lines 14–16 in Box 8). We repeated the code in Box 8 100 times and generated 100 random partitions of the data into training and testing sets. Each partition renders an estimate of prediction accuracy for each of the models.

**Box 8 Correlation between y and $\hat{y}$ for training and validation data sets**

**Table:** 

1	yNA = y
2	seed = 12345; set.seed(seed)
3	#randomly masking one environment for each variety
4	whichNa = seq(from = 0,to = 2392,by = 4)+sample(1:4,size = 599,replace = T)
5	yNA[whichNa]=NA
6	OLS = FW(y = yNA,VAR = VAR,ENV = ENV, method=”OLS”)
7	GibbsI = FW(y = yNA,VAR = VAR,ENV = ENV,
8	method=”Gibbs”,seed = seed,nIter = 50000, burnIn = 5000)
9	GibbsA = FW(y = yNA,VAR = VAR,ENV = ENV,
10	method=”Gibbs”,A = wheat.G,seed = seed,nIter = 50000,burnIn = 5000)
11	cor(y[-whichNa],OLS$yhat[-whichNa,])
12	cor(y[-whichNa],GibbsI$yhat[-whichNa,])
13	cor(y[-whichNa],GibbsA$yhat[-whichNa,])
14	cor(y[whichNa],OLS$yhat[whichNa,])
15	cor(y[whichNa],GibbsI$yhat[whichNa,])
16	cor(y[whichNa],GibbsA$yhat[whichNa,])

The mean correlation (of the 100 replicates) between phenotypes and predictions in the training data set (*i.e.*, for the entries of y that did not have missing values) follows the same patterns as in Example 1, where OLS fitted the data best: 0.95 for OLS, 0.89 for GibbsI, and 0.86 for GibbsA. However, the mean prediction correlation (of the 100 replicates) for the entries of the validation set has reversed orders: 0.61 for OLS, 0.77 for GibbsI, and 0.80 for GibbsA.

**Figure 5:**
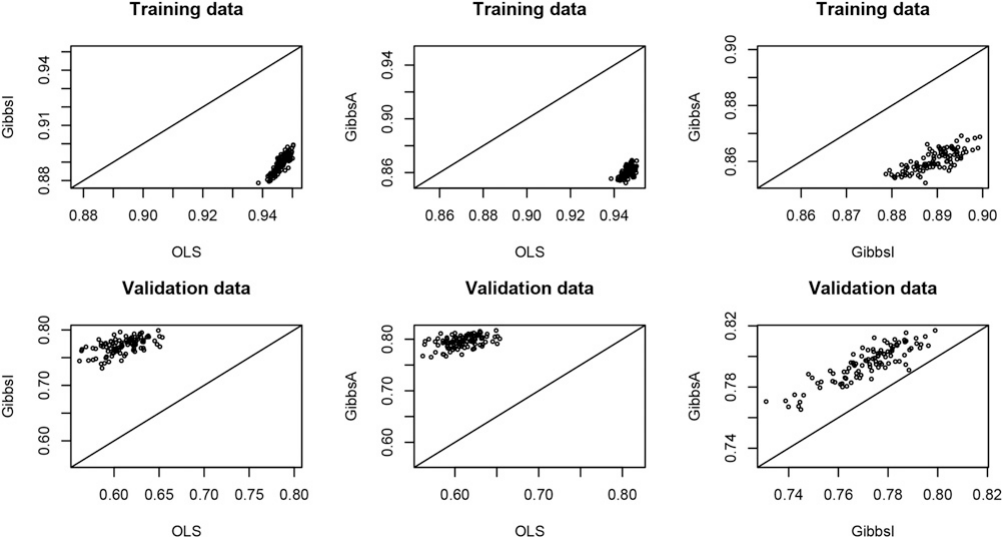
Prediction accuracy for training and validation sets for the three methods implemented in Box 8.

In Figure 5, we plotted the estimated prediction correlation between predictions and observations in training (1st row of plots) and testing (2nd row of plots) data sets. Plots in the 1st, 2nd, and 3rd column correspond to comparisons of: OLS *vs.* GibbsI, OLS *vs.* GibbsA, and GibbsI *vs.* GibbsA, respectively. Within each plot each point represents the accuracy obtained in a partition for the models represented in the vertical and horizontal axis. Points above (below) the 45° line indicate higher (lower) accuracy of the model in the vertical axis, relative to the one in the horizontal axis. We observed that OLS always fitted the data better than GibbsI and GibbsA in the training data sets; however, GibbsI and GibbsA always outperformed OLS by a sizable margin in terms of prediction accuracy in testing data sets. Finally, incorporating genetic information (GibbsA) always led to higher prediction accuracy than models that assumed independence between lines (GibbsI).

We also noted in Table 4 that the correlations (here we reported results only for the first replicate) for the parameter estimates among different models reduced compared to Example 1 due to the missing values. For example, the correlation for the estimated **b** among different models reduced to 0.85 between OLS and GibbsI, 0.64 between OLS and GibbsA, and 0.79 between GibbsI and GibbsA.

**Table 4:** Pearson’s product-moment correlation between parameter estimates derived by each of the three methods implemented in Box 8 (results from the first replicate only)

	OLS–GibbsI	OLS–GibbsA	GibbsI–GibbsA
$\hat{h}$	1.00	1.00	1.00
$\hat{b}$	0.85	0.64	0.79
$\hat{g}$	0.96	0.73	0.77
$\hat{y}$	0.91	0.87	0.97

## Computation Time for 599 Wheat Lines

We ran the FW function in an Intel Core i7 1867 MHz Processor (R was executed in a single thread) with 16 GB of RAM memories. We recorded the memory and time usage for Gibbs methods with 50000 iterations. With the full data set (599 varieties, 2396 observations) the process used approximately 50 M of RAM memory for GibbsA, 17 M of RAM for GibbsI, and 153 M for OLS. The time needed to finish the process was: 11 min for GibbsA, 3 min for GibbsI, and 2 sec for OLS.

## Concluding Remarks

The FW package allows fitting Finlay–Wilkinson regression with ordinary least square method and Bayesian method. For Bayesian method, covariance matrix among varieties and environments can be included in the model. The interface allows the user to fit the models (*e.g.*, OLS *vs.* Gibbs) and visualize the results easily. The algorithms for Gibbs sampler are implemented in C and the speed is high. The package also provided flexibility for changing the hyper-parameters and model output.

For incomplete/unbalanced experimental design the Bayesian approach is expected to have better statistical performance and prediction accuracy than the traditional two-step OLS method. Furthermore, the Bayesian models implemented in FW allows incorporating pedigree and marker information as well as modeling environment covariance. A cross-validation study based on real wheat data confirmed those expectations; indeed, the Bayesian method incorporating relationships between lines had a prediction accuracy that was 30% greater than the two-step OLS method.

## Supplementary Material

Supporting Information
