# Supplementary material for: FW: An R Package for Finlay–Wilkinson Regression that Incorporates Genomic/Pedigree Information and Covariance Structures Between Environments
Source: G3 (Bethesda). 2015 Dec 29;6(3):589–97. doi: 10.1534/g3.115.026328 (PMC4777122; doi:10.1534/g3.115.026328)
Supplement: Supporting Information [file supp_6_3_589__index.html]

FW: An R Package for Finlay–Wilkinson Regression that Incorporates Genomic/Pedigree Information and Covariance Structures Between Environments — Supporting Information 

# FW: An R Package for Finlay–Wilkinson Regression that Incorporates Genomic/Pedigree Information and Covariance Structures Between Environments

## Supporting Information for Lian and de los Campos, 2016

**Files in this Data Supplement:**

- File S1 - Implementation of Gibbs Sampler. (.pdf, 192 KB)
- File S2 - 1.Treatment of missing values. (.pdf, 375 KB)
