# Supplementary material for: FW: An R Package for Finlay–Wilkinson Regression that Incorporates Genomic/Pedigree Information and Covariance Structures Between Environments
Source: G3 (Bethesda). 2015 Dec 29;6(3):589–97. doi: 10.1534/g3.115.026328 (PMC4777122; doi:10.1534/g3.115.026328)
Supplement: Supporting Information [file supp_g3.115.026328_FileS1.pdf]

File S1

Implementation of Gibbs Sampler

Full conditional distributions

Gibbs sampler is the algorithm to sample each unknown variable based on their full conditional distributions. For each iteration of Gibbs sampler, we sample each parameter from their full conditional distributions and iterate through all the parameters. To present the full conditional distribution for all the parameters, we first re-express the model in matrix form.

$$\mathbf{y} = \mathbf{1}\mu + \mathbf{Z_g}\,\mathbf{g} + \mathbf{Z_h}\,\mathbf{h} + (\mathbf{Z_g}\,\mathbf{b})\#(\mathbf{Z_h}\,\mathbf{h}) + \boldsymbol{\varepsilon}$$

$$= \mathbf{1}\mu + \mathbf{Z_g}\mathbf{L_A}\boldsymbol{\delta}_\mathbf{g} + \mathbf{Z_h}\,\mathbf{L_H}\boldsymbol{\delta}_\mathbf{h} + (\mathbf{Z_g}\,\mathbf{L_A}\boldsymbol{\delta}_\mathbf{b})\#[\mathbf{Z_h}\,\mathbf{L_H}\boldsymbol{\delta}_\mathbf{h}\,] + \boldsymbol{\varepsilon}$$

Where # represents element-wise product between two vectors, **y** is a vector of observed phenotypes, **g** is a vector for the main genotype effects, $\mathbf{Z_g}$ is the incidence matrix that relates each level of **g** to **y**, **h** is a vector for the main environment effects, $\mathbf{Z_h}$ is the incidence matrix that related each level of **h** to **y**, **b** is the regression of the varieties on the main environmental effect. Instead of sampling **b**, **g**, **h** directly, we transform them into an orthogonal basis as $\boldsymbol{\delta}_\mathbf{g} = \mathbf{L_A}^{-1}\mathbf{g}$, $\boldsymbol{\delta}_\mathbf{b} = \mathbf{L_A}^{-1}\mathbf{b}$ and $\boldsymbol{\delta}_\mathbf{h} = \mathbf{L_H}^{-1}\mathbf{h}$ where $\mathbf{L_A}$ and $\mathbf{L_H}$ are lower triangular matrix from the Cholesky decomposition of **A** and **H** ($\mathbf{L_A}\mathbf{L_A}' = \mathbf{A}$, $\mathbf{L_H}\mathbf{L_H}' = \mathbf{H}$). The $\boldsymbol{\delta}_\mathbf{g}$, $\boldsymbol{\delta}_\mathbf{b}$ and $\boldsymbol{\delta}_\mathbf{h}$ all follow IID Normal distribution: $\boldsymbol{\delta}_\mathbf{g} \sim \mathrm{N}(\mathbf{0}, \mathbf{I}\sigma_g^2)$, $\boldsymbol{\delta}_\mathbf{b} \sim \mathrm{N}(\mathbf{0}, \mathbf{I}\sigma_b^2)$ and $\boldsymbol{\delta}_\mathbf{h} \sim \mathrm{N}(\mathbf{0}, \mathbf{I}\sigma_h^2)$.

The overall mean μ is assigned a flat prior. The scale parameters all follow a scaled inverse chi-square distribution: $\sigma_\varepsilon^2 \sim \chi^{-2}(\nu_\varepsilon, S_\varepsilon^2)$, $\sigma_g^2 \sim \chi^{-2}(\nu_g, S_g^2)$, $\sigma_b^2 \sim \chi^{-2}(\nu_b, S_b^2)$, $\sigma_h^2 \sim \chi^{-2}(\nu_h, S_h^2)$. In the parameterization used we have $E[\sigma^2] = \frac{\nu S^2}{\nu-2}$.

General results for conjugate Normal distribution

We define a model with the general form $\mathbf{y} = \mathbf{X\beta} + \boldsymbol{\varepsilon}$, and with prior distributions $\boldsymbol{\beta} \sim \mathrm{N}(\mathbf{0}, \mathbf{I}\sigma_\beta^2)$, $\boldsymbol{\varepsilon} \sim \mathrm{N}(\mathbf{0}, \mathbf{I}\sigma_\varepsilon^2)$, $\sigma_\varepsilon^2 \sim \chi^{-2}(\nu_\varepsilon, S_\varepsilon^2)$ and $\sigma_\beta^2 \sim \chi^{-2}(\nu_\beta, S_\beta^2)$. When sampling a location parameter β_i in the model, all other parameters will be treated as known. Let $\tilde{\mathbf{y}} = \mathbf{y} - \mathbf{X}_{-\mathbf{i}}\boldsymbol{\beta}_{-i}$=$\mathbf{X}_\mathbf{i}\beta_i + \boldsymbol{\varepsilon}$, where ($-i$ means all other columns (elements) except column (element) i). The conditional distribution of the new data vector $\tilde{\mathbf{y}}$ will be $p(\tilde{\mathbf{y}}|\mathbf{X}_\mathbf{i}\beta_i) \sim \mathrm{N}(\mathbf{X}_\mathbf{i}\beta_i, \mathbf{I}\sigma_\varepsilon^2)$. The full conditional posterior distribution for β_i, σ_ε^2 and σ_β^2 will be:

$p(\beta_i|\mathrm{ELSE}) \sim \mathrm{N}(C^{-1}r, C^{-1})$, where $C = \frac{\mathbf{X_i}'\mathbf{X_i}}{\sigma_\varepsilon^2} + \frac{1}{\sigma_\beta^2}$ and $r = \frac{\mathbf{X_i}'\tilde{\mathbf{y}}}{\sigma_\varepsilon^2}$

$p(\sigma_\varepsilon^2|\mathrm{ELSE}) \sim \chi^{-2}(n + \nu_\varepsilon, \frac{\nu_\varepsilon S_\varepsilon^2 + \boldsymbol{\varepsilon}'\boldsymbol{\varepsilon}}{n+\nu_\varepsilon})$, where n is the number of observations

$p(\sigma_\beta^2|\mathrm{ELSE}) \sim \chi^{-2}(q_\beta + \nu_\beta, \frac{\nu_\beta S_\beta^2 + \boldsymbol{\beta}'\boldsymbol{\beta}}{q_\beta+\nu_\beta})$, where q_β is the number of levels in $\boldsymbol{\beta}$.

Therefore, to sample a single location parameter from the conjugate Normal distribution, all we need to know is the incidence matrix $\mathbf{X_i}$ and the form of C. We can use the above rules to express the full conditional distributions for the unknown parameters. In Table 1, we listed the $\mathbf{X_i}$ and C for each of the location parameters for μ, δ_{h_i}, δ_{b_i} and δ_{g_i}.

Table 1 The $\mathbf{X_i}$ and C needed to obtain the full conditional distributions for μ, δ_{h_i}, δ_{b_i} and δ_{g_i}.

Parameters	$\mathbf{X_i}$	C
μ	$\mathbf{1}$	$\frac{\mathbf{1}'\mathbf{1}}{\sigma_\varepsilon^2}$

δ_{h_i}	$\mathbf{Z_h L_{H_i}} \# (\mathbf{Z_g b} + \mathbf{1})$	$\frac{\mathbf{X_i}'\mathbf{X_i}}{\sigma_\varepsilon^2} + \frac{1}{\sigma_h^2}$
δ_{b_i}	$(\mathbf{Z_h h}) \# (\mathbf{Z_g L_{A}}_i)$	$\frac{\mathbf{X_i}'\mathbf{X_i}}{\sigma_\varepsilon^2} + \frac{1}{\sigma_h^2}$
δ_{g_i}	$\mathbf{Z_g L_{A}}_i$	$\frac{\mathbf{X_i}'\mathbf{X_i}}{\sigma_\varepsilon^2} + \frac{1}{\sigma_g^2}$

The full conditional distributions for the variance components will be:

$p(\sigma_\varepsilon^2|\text{ELSE}) \sim \chi^{-2}(n+\nu_\varepsilon, \frac{\nu_\varepsilon S_\varepsilon^2 + \boldsymbol{\varepsilon}'\boldsymbol{\varepsilon}}{n+\nu_\varepsilon})$, $\sigma_g^2|\text{ELSE} \sim \chi^{-2}(q_g+\nu_g, \frac{\nu_g S_g^2 + \boldsymbol{\delta}_\mathbf{g}'\boldsymbol{\delta}_\mathbf{g}}{q_g+\nu_g})$, $\sigma_b^2|\text{ELSE} \sim \chi^{-2}(q_g+\nu_b, \frac{\nu_b S_b^2 + \boldsymbol{\delta}_b'\boldsymbol{\delta}_b}{q_g+\nu_b})$ and $\sigma_h^2|\text{ELSE} \sim \chi^{-2}(q_h+\nu_h, \frac{\nu_h S_h^2 + \boldsymbol{\delta}_h'\boldsymbol{\delta}_h}{q_h+\nu_h})$, where q_g is the number of levels for the varieties, and q_h is the number of levels for the environments.

Implementation of Gibbs Sampler

For each iteration of Gibbs Sampler, we sample each parameter from their full conditional distributions, the detailed implementation is as following:

1. Set initial values for all the parameters.

2. Set initial values for missing values of **y** (denote here as $\mathbf{y}_{\text{NA}}$).

3. Sample δ_{h_i}, and update $\boldsymbol{\varepsilon}$. Do this for all i from 1 to q_h.

4. Sample δ_{b_i} and update $\boldsymbol{\varepsilon}$; sample δ_{g_i} and update $\boldsymbol{\varepsilon}$. Do this for all i from 1 to q_g.

5. Sample σ_ε^2, σ_h^2, σ_b^2 and σ_g^2 from their full conditional distributions.

6. Sample μ and update $\boldsymbol{\varepsilon}$.

7. Update $\hat{\mathbf{y}}$=$\mathbf{y} - \boldsymbol{\varepsilon}$.

8. Sample $\mathbf{y}_{\mathrm{NA}}$ from $\mathrm{N}(\hat{\boldsymbol{y}}_{\mathrm{NA}}, \mathbf{I}\sigma_{\varepsilon}^{2})$. Update $\mathbf{y}_{\mathrm{NA}}$ and $\boldsymbol{\varepsilon}$.

9. Repeat steps 2 through 8 for the number of specified iterations.

References

Su, G., P. Madsen, M. S. Lund, D. Sorensen, I. R. Korsgaard, and J. Jensen. 2006. Bayesian analysis of the linear reaction norm model with unknown covariates. Journal Of Animal Science 84:1651-1657.
