# Supplementary material for: FW: An R Package for Finlay–Wilkinson Regression that Incorporates Genomic/Pedigree Information and Covariance Structures Between Environments
Source: G3 (Bethesda). 2015 Dec 29;6(3):589–97. doi: 10.1534/g3.115.026328 (PMC4777122; doi:10.1534/g3.115.026328)
Supplement: Supporting Information [file supp_g3.115.026328_FileS2.pdf]

File S2

1. Treatment of missing values

The FW function support missing values on the response, but the treatment is different in the OLS and Bayesian methods.

Ordinary-least squares method. For OLS regression, missing values are dropped; therefore, if all the entries corresponding to a genotype or to an environment are NA, the corresponding estimates of effects are reported as NA.

Bayesian method. In this case the entries that have NA are not removed, instead, in each iteration of the Gibbs sampler the un-observed missing values are sampled from the corresponding fully conditional density. If data from a genotype or environment are all NAs, the Gibbs sampler can still render estimate about the parameters of these genotypes or environments, but these estimates are entirely based on the co-variance between the genotype or environment and other genotypes (**A**) or environments (**H**). This allows users to infer intercepts and slopes of varieties that may have not been evaluated in field trials, provided that such varieties are correlated with others that have data via **A**. Similarly, one could predict performance of varieties in environments with limited or no data, provided that this environment is correlated with other for which data is available. The user should not specify models with genotypes or environment with complete missing values (data from a genotype or environment are all NAs) if **A** or **H** are diagonal.

2. Hyper-parameter setup

In the main article, we mentioned that the prior distributions for σ_ε^2, σ_g^2, σ_b^2 and σ_h^2 are all set to be scaled-inverse χ^2 distributions: $\sigma_\varepsilon^2 \sim (\nu_\varepsilon, S_\varepsilon^2)$, $\sigma_g^2 \sim$

$\chi^{-2}(\nu_g, S_g^2), \sigma_b^2 \sim \chi^{-2}(\nu_b, S_b^2), \sigma_h^2 \sim \chi^{-2}(\nu_h, S_h^2)$. The set of hyper-parameter that control the shape of these prior distributions are the degrees of freedom (ν_ε, ν_g, ν_b and ν_h) and scale parameters (S_ε^2, S_g^2, S_b^2 and S_h^2).

The default setup was similar as discussed in (Pérez et al., 2010). Small values of degree of freedom (ν_ε, ν_g, ν_b, ν_h) for the scaled inverse chi-square distributions are generally selected. The default is $\nu_\varepsilon = \nu_g = \nu_b = \nu_h = 5$, but the user can choose any small positive numbers.

The scale parameter S^2 is set to meet the mode of density: mode (σ^2) = $\frac{\nu S^2}{\nu+2}$. If we have some prior estimate of σ^2 as $\hat{\sigma}^2$, we will have $S^2 = \hat{\sigma}^2 \frac{\nu+2}{\nu}$. By default, the FW package sets the prior estimates of σ_ε^2, σ_g^2, σ_b^2 and σ_h^2 as $\hat{\sigma}_\varepsilon^2 = 0.5\ \hat{\sigma}_p^2$, $\hat{\sigma}_g^2 = 0.25\hat{\sigma}_p^2$, $\hat{\sigma}_b^2 = \hat{\sigma}_h^2 = 0.5\ \hat{\sigma}_p$, where $\hat{\sigma}_p^2$ is the estimate of the total phenotypic variance. When assuming independent random effects, the variance of the interaction term will be $\sigma_b^2\sigma_h^2$. Therefore, setting $\hat{\sigma}_b^2 = \hat{\sigma}_h^2 = 0.5\ \hat{\sigma}_p$ will lead to a $0.25\hat{\sigma}_p^2$ interaction variance. The scale parameters can then be set up as $S_\varepsilon^2 = \frac{\hat{\sigma}_\varepsilon^2(\nu_\varepsilon+2)}{\nu_\varepsilon}$, $S_g^2 = \frac{\hat{\sigma}_g^2(\nu_g+2)}{\nu_g}$, $S_b^2 = \frac{\hat{\sigma}_b^2(\nu_b+2)}{\nu_b}$ and $S_h^2 = \frac{\hat{\sigma}_h^2(\nu_h+2)}{\nu_h}$.

The FW package provides arguments to setup hyper parameters: `dfe`, `dfg`, `dfb`, `dfh` will set up the degrees of freedom ν_ε, ν_g, ν_b and ν_h respectively; `priorVARe`, `priorVARg`, `priorVARb`, `priorVARh` will setup the prior estimates for σ_ε^2, σ_g^2, σ_b^2 and σ_h^2; the scale parameter S^2 is then automatically reset based on the mode of scaled inverse χ^2 distribution. For example, if we set `dfe=5`, and `priorVARe=0.2`, then S_ε^2

will be automatically set to be `priorVAR*(dfe+2)/dfe=0.2*(5+2)/5=0.28`. The code in Box S1 shows an example of setting user-defined hyper-parameters.

Box S1: Changing hyper parameters in FW.

```
lm1=FW(y=yNA,VAR=VAR,ENV=ENV,dfe=1,dfg=1,dfb=1,dfh=0.1,
priorVARe=0.36,priorVARg=0.18,priorVARh=0.74,priorVARb=0.18)
```

3. Initial values and assessment of convergence with multiple chains

The FW package sets default initial values for all the chains. The default initial values for chain 1 are set as $\mu = 0$, **g**=**0**, **b**=**0**, **h**=**0**, σ_ε^2 =0.5 $\hat{\sigma}_p^2$, $\sigma_g^2 = 0.25\hat{\sigma}_p^2$, $\sigma_b^2 = \sigma_h^2$ 0.5$\hat{\sigma}_p$, where $\hat{\sigma}_p^2$ is the estimate of the total phenotypic variance. The default initial values for all other chains are generated from random sampling: μ from N(0, 0.5$\hat{\sigma}_p^2$), **g** from N(**0**, 0.25$\hat{\sigma}_p^2$**I**), **b** and **h** from N(**0**, 0.5$\hat{\sigma}_p$**I**), the variance components are generated from uniform distribution whose boundaries are set as half and twice the values used in chain 1: σ_ε^2 from 0.5$\hat{\sigma}_p^2$U(0.5, 2) , σ_g^2 from 0.25$\hat{\sigma}_p^2$U(0.5, 2), σ_b^2 and σ_h^2 from 0.5$\hat{\sigma}_p$U(0.5, 2).

The FW package provided the `inits` argument to set user defined initial values for Gibbs sampler. The parameters taken by R (the corresponding math representation in in parentheses) are: `mu` (μ), `g` (**g**), `b` (**b**), `h`(**h**), `var_e` (σ_ε^2), `var_g` (σ_g^2), `var_b` (σ_b^2), `var_h` (σ_h^2). These values must be supplied in a named list as `inits=list(inits1=list(mu=,g=,b=,h=,var_e=,var_g=,var_b=,var_h=),inits2=list(...),inits3=NULL)`. If any of the chains is set to be `NULL`, the initial values for that chain will be set up with default initial values from random sampling.

The example code in Box S2 fitted a FW model with two chains for Gibbs sampler. Line 1-4 of Box S2 secified the initial values for the two chains: the first chain took the user-defined initial values, and by setting `inits2=NULL`, the second chain would have default initial values from random sampling. Line 6-9 in Box S2 fitted the model `fit2_50k` with two chains and generated a trace plot for `h[1]` and `h[2]`. From Figure S1 we can see that the two chains are mixing reasonably well.

Box S2: Fitting more than one chain with user defined initial values.

```
1 ng=length(unique(VAR));nh=length(unique(ENV));
2
3 inits=list(inits1=list(mu=0,g=rep(0,ng),b=rep(0,ng),h=rep(0,nh),
4 var_e=0.36, var_g=0.18,var_b=0.18,var_h=0.74),inits2=NULL)
5
6 fit2_50k=FW(y=yNA,VAR=VAR,ENV=ENV,nchain=2, seed=c(12345,12346),
7 inits=inits, saveAt= "fit2_50k",nIter=50000, burnIn=5000)
8 load("fit2_50ksamps.rda")
9 plot(samps[,c("h[1]", "h[2]")])
```

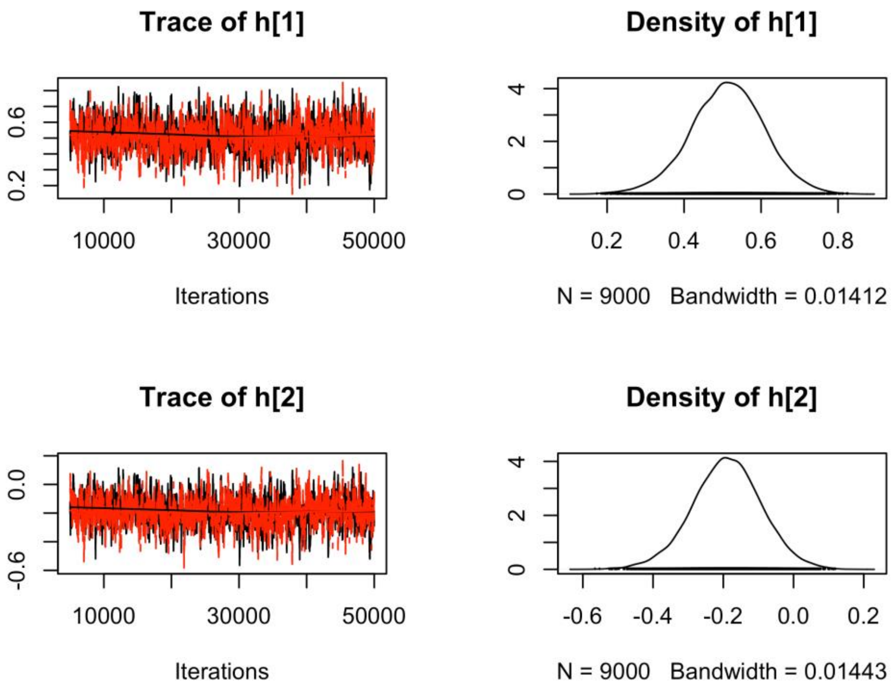

Figure S1. Trace and density plot for `h[1]` and `h[1]` for two chains.

References

Pérez, P., G. de Los Campos, J. Crossa, and D. Gianola. 2010. Genomic-enabled prediction based on molecular markers and pedigree using the bayesian linear regression package in R. The Plant Genome 3: 106-116.
